# Effects over time of self-reported direct and vicarious racial discrimination on depressive symptoms and loneliness among Australian school students

**DOI:** 10.1186/s12888-017-1216-3

**Published:** 2017-02-03

**Authors:** Naomi Priest, Ryan Perry, Angeline Ferdinand, Margaret Kelaher, Yin Paradies

**Affiliations:** 1 ANU Centre for Social Research and Methods, Australian National University, Beryl Rawson Building (Building 13) Rm 2.30, Acton, ACT 2610 Australia; 2 Centre for Health Equity, Melbourne School of Population and Global Health, University of Melbourne, Carlton, VIC 3010 Australia; 3 Centre for Health Policy, Melbourne School of Population and Global Health, University of Melbourne, Carlton, VIC 3010 Australia; 4 Alfred Deakin Institute for Citizenship and Globalisation, Deakin University, 221 Burwood Hwy, Burwood, VIC 3125 Australia

**Keywords:** Racial discrimination, Mental health, Depression, Loneliness, School

## Abstract

**Background:**

Racism and racial discrimination are increasingly acknowledged as a critical determinant of health and health inequalities. However, patterns and impacts of racial discrimination among children and adolescents remain under-investigated, including how different experiences of racial discrimination co-occur and influence health and development over time. This study examines associations between self-reported direct and vicarious racial discrimination experiences and loneliness and depressive symptoms over time among Australian school students.

**Methods:**

Across seven schools, 142 students (54.2% female), age at T1 from 8 to 15 years old (M = 11.14, SD = 2.2), and from diverse racial/ethnic and migration backgrounds (37.3% born in English-speaking countries as were one or both parents) self-reported racial discrimination experiences (direct and vicarious) and mental health (depressive symptoms and loneliness) at baseline and 9 months later at follow up. A full cross-lagged panel design was modelled using MPLUS v.7 with all variables included at both time points.

**Results:**

A cross-lagged effect of perceived direct racial discrimination on later depressive symptoms and on later loneliness was found. As expected, the effect of direct discrimination on both health outcomes was unidirectional as mental health did not reciprocally influence reported racism. There was no evidence that vicarious racial discrimination influenced either depressive symptoms or loneliness beyond the effect of direct racial discrimination.

**Conclusions:**

Findings suggest direct racial discrimination has a persistent effect on depressive symptoms and loneliness among school students over time. Future work to explore associations between direct and vicarious discrimination is required.

## Background

Globally across contexts and populations racism and racial discrimination are recognized as critical determinants of health and health inequalities [41, 65]. The added burden of racism is arguably the most critical distinctive social exposure influencing health experienced by racially stigmatized groups globally, including indigenous peoples, ethnic minorities and in some cases migrants [66]. Interpersonal racism, that is racism that is personally mediated via the expression of prejudicial attitudes and discriminatory behavior, is a psychosocial stressor that adversely affects a broad range of health outcomes and health risk behaviors as documented in several recent meta-analyses [33, 38, 40, 52]. However, this evidence is largely focused on adult populations, with knowledge of patterns and health impacts of racial discrimination among children and adolescents far less understood [37, 45, 52].

Moreover, children and young people are considered particularly vulnerable to the harmful effects of racial discrimination as a psychosocial stressor [37, 45]. There is growing scientific consensus that childhood adversity and stress influence health profoundly both in childhood and later in life, including physical and mental health and cardiovascular, metabolic and immune function [54]. Two key ways in which early experiences can influence adult health are proposed - by repeated exposure to stress that accumulates damage over time or by the biological embedding of stress and adversities during sensitive developmental periods [28, 30, 54]. However, increased understanding of potentially malleable childhood risk factors is needed, including patterns of different forms of childhood stressors such as racial discrimination, and how they influence health over time [54].

Existing racial discrimination research among children and adolescents has concentrated mostly on direct discrimination experiences where children and adolescents themselves are the targets [42, 45, 46]. Vicarious (or indirect) forms of racial discrimination, that is hearing about or seeing another person’s experience of racism ([34]; Harrell 25) as well as carers or close family members experiencing discrimination that may or may not be witnessed by children and adolescents [44] are now also starting to be documented in relation to negative child and adolescent outcomes. This is consistent with considerable evidence demonstrating harmful effects for children and youth of vicarious, indirect experiences of violence such as hearing reports of violence against people they know or witnessing violence [63]. Witnessing bullying and harassment has also been shown to impact negatively on mental health for children and adolescents [36, 51]. However, key knowledge remains nascent regarding the impacts of vicarious racial discrimination for children and adolescents. Current work on vicarious racism and health and development outcomes for children [6, 9, 20, 22, 29, 44, 57] and adolescents [8, 15, 19] almost exclusively focuses on carer reports of racism rather than child and adolescent reports of vicarious experiences (although, see [42, 46]). Moreover, the co-occurrence and impacts of direct and vicarious racial discrimination experiences reported by children and adolescents over time have, so far, not been investigated empirically [42, 46].

Most studies of racial discrimination and child and youth health have examined negative mental health outcomes, particularly reporting significant associations with childhood depression [45]. Within this body of work, loneliness is examined as a particular depressive symptom [10] as well as a separate outcome [26, 35], with experiences of racial discrimination associated with both depressive symptoms and loneliness among young populations [35, 67]. However, more work is needed using longitudinal designs to understand the specific effects of racial discrimination, both direct and vicarious, on these two closely related mental health outcomes over time [42, 45, 46].

Increased understanding of the patterns and impacts of different types of experiences of racial discrimination on children and adolescents within the key settings of their lives is thus an important research priority. One such critical setting is school, where children and adolescents spend much of their time interacting with peers [24]. Schools are complex multi-level institutions that impact children’s development in a variety of ways, including teacher, peer and physical environmental influences [14]. As such, they are central settings for interpersonal relationships, including racism as expressed through racial discrimination, racial bullying and racial victimization [7].

While empirical research on racism and child and adolescent health has been growing in recent years, a recent systematic review of the field revealed most studies have been conducted in the United States [45] with work emerging in countries where ethnic diversity is largely driven by recent migration, such as the United Kingdom [3], Canada [21], Australia [44] and New Zealand [11] as well as nations with longer histories of cultural diversity such as the Netherlands [2, 60].

Australia has a high level of racial/ethnic diversity among its primary and secondary school student population with one third of students either immigrants themselves or born in Australia to at least one immigrant parent [27]. Another 4.9% are Indigenous [4]. More than 230 countries of origin and over 200 languages and dialects are represented; from 2006 to 2010, 17.5% of permanent additions to the Australian population aged 0–17 years were from Southeast Asia, 17.4% from Southern Asia, 12.0% from Southern and East Africa, 12.0% from Northeast Asia and 5.2% from the Middle East (Department of Immigration and Border Protection 2010). In this context of high and increasing levels of racial ethnic diversity, racism and racial discrimination are substantial concerns for many Australian students. Yet research on the prevalence and impact of racist experiences among these students is limited. A 2009 survey of 698 secondary students across four states found 70% of those from non-Anglo backgrounds reporting experiences of racism during their lifetime, with 67% of these experiences occurring in school [34]. More recently, a survey of 263 primary and secondary students from diverse racial/ethnic backgrounds in Victoria, Australia, found high levels of perceived racial discrimination with at least one form of racism experienced directly by 32.2% of the sample monthly or more, and by 22.1% every day [42, 46].

Previously we have reported direct experiences of racial discrimination as robustly associated with higher loneliness and depressive symptoms among Australian students cross-sectionally, and that the association with depressive systems was attenuated to marginal significance for students with low motivated fairness, that is low levels of motivation to respond without prejudice [42, 46]. However, whether these patterns of association are maintained over time, or the direction of association between perceived discrimination and loneliness and depressive symptoms is unidirectional, is as yet unknown. As noted, previous cross-sectional studies assume a direction of effects in which discrimination precedes poor mental health but have not examined the simultaneous effects of direct and vicarious racism on mental health over time in a full cross-lagged model. Furthermore, previous research has not distinguished between discrete mental health dimensions of loneliness and depressive symptoms over time in this age group.

Depressive symptoms and loneliness are related dimensions of mental health and psychosocial functioning, and are conceptualised and measured using various means among children and adolescents as well as adults [32, 47]. For example, several depression scales for children include ‘feeling lonely’ as a domain [12, 16]. However, more recent work identifies that loneliness and depressive symptoms, are conceptually distinct forms, though partly overlapping constructs [55, 61]. Loneliness and depressive symptoms have been shown to have different relations with other constructs, including various aspects of adjustment, different developmental trajectories, and different patterns by gender implying further distinction between them and highlighting the need to assess both loneliness and depressive symptoms [55]. Loneliness and depressive symptoms as reciprocally associated, and potentially reinforcing one another over time, has also been empirically shown among adolescents [61].

As recommended [55, 61], in this study we consider depressive symptoms and loneliness as two separate constructs among students, and examine the effects of racial discrimination on each construct over time, as well as stability of, and associations between, each construct modelled simultaneously over time in a full cross-lagged model. The present study aims to examine the auto-regressive and cross-lagged associations between perceived racial discrimination and loneliness and depressive symptoms over time among Australian primary and secondary school students. Using a longitudinal model, the study will be able to determine over time (a) the extent to which mental health symptoms (i.e., depressive symptoms and loneliness) are determined by their pre-existing levels, versus (b) the independent effect of racial discrimination (direct and vicarious) on each of these symptoms.

We hypothesize that racial discrimination (direct and vicarious) independently predict both depressive symptoms and loneliness, even after accounting for pre-existing levels of depressive symptoms and loneliness.

These aims were investigated using one component of data collected for the evaluation of the Localities Embracing and Accepting Diversity (LEAD) program. LEAD was a community-based intervention to counter racial discrimination and promote racial/ethnic diversity across multiple settings in two local government areas (LGAs) in Victoria, Australia; primary and secondary schools being one site of intervention [17, 42, 46]. This provided a unique opportunity to examine the effects of discrimination on mental wellbeing across both primary and secondary school students over time within a diverse community setting.

## Methods

### Design and setting

Participants were students across seven schools in two LGAs; three primary and two secondary schools from LGA A and two primary and two secondary schools from LGA B. LGAs nominated themselves to be intervention sites as part of a competitive process facilitated by the funding organisations and were not selected due to particularly high levels of racial discrimination in comparison to other Victorian communities. The populations of both LGAs have high levels of cultural and linguistic diversity and low to medium-average socio-economic status. LGA A is a large regional town with a population of approximately 60,000 people located about 200 km from Melbourne while LGA B is an outer suburban area of Melbourne (population approximately 155,000). After being recruited by LGA staff, schools developed and implemented intervention strategies supported by LGA staff. Most commonly, schools conducted pro-diversity training with staff and held shared cultural days with students and families that included a mix of arts-based activities and cooperative games that highlighted human rights principles of respect and dignity.

### Procedure and sample

One hundred forty two school students completed surveys both at Time 1 (T1) and 9 months later at Time 2 (T2). There were 65 (45.8%) males and 77 (54.2%) females with a mean age at Time 1 of 11.14 years (*SD* = 2.20; ranging from 8 to 15 years). There were 53 students (37.3%) born in English-speaking countries who also had one or both parents born in English speaking countries; 46 students (32.4%) born in English-speaking countries who had parents born in non-English speaking countries, 43 (30.3%) who were born in non-English speaking countries themselves as well as their parents. A further eight students did not know where one or both of their parents were born or had missing data and were excluded from analyses.

Data were collected from students in participating schools at baseline (T1) and 9 months later at follow up (T2) via self-report surveys with parental consent for students as previously described [42, 46]. Parent consent forms and study information was translated into languages other than English and distributed via multicultural education aides in each school when relevant to ensure parents were able to provide informed consent. All students had sufficient skills to complete surveys in English. One of the authors guided primary school students through the survey as a group, while secondary school students completed it independently with an author available for questions.

### Measures

#### Demographic variables

Students reported age, gender, and school year level. Following Priest et al. [46], three birth-country categories were created by categorising open-ended responses as (1) students who were born, and whose parents were born, in an English-speaking country (reference group); (2) students who were born in an English-speaking country and whose parents were born in non-English speaking country (minority English); and (3) students who were born in a non-English-speaking country whose parents were also born in a non-English-speaking country (minority non-English). These categories reflect the Australian context where self-reported race and ethnicity is not routinely collected in administrative data, nor are these concepts part of the community vernacular. Cultural background, country of birth and language spoken at home, as well as Indigenous identification, are instead used in various combinations within Australian data collection and reporting. A full list of countries of birth by the analytic categories used is provided in Appendix.

#### Experiences of racial discrimination

Racial discrimination was assessed via seven items examining direct experiences (*Has this happened to YOU at school?: Other students said you don’t belong in Australia; Other students didn’t want to play with you because of your culture; A teacher thought you couldn’t do something because of your culture; You were left out by a student because of your culture; You were left out by a teacher because of your culture; You were teased or called names by other students because of your culture; You were spat on, pushed or hit by other students because of your culture*), and three items examining vicarious experiences toward other students (*Has this happened to OTHER students at your school?: Students are left out because of their cultural group; Students are called names or teased because of their cultural group; Students are spat on, pushed or hit because of their cultural group*). These items were developed by the authors with reference to systematic reviews on best-practice approaches to measuring experiences of racism among both adults [5, 53] and children [45]. Existing measures of perceived racial discrimination [5, 45, 53] were unsuitable for this study as they were designed for adults or adolescents only, lacked sufficient psychometric validity, were designed for specific racial/ethnic groups, or were too lengthy (i.e., >20 items) as the funding and wider context of the evaluation study limited the overall survey length to less than 40 items in total. Factor analysis of these experiences of racial discrimination items has been reported elsewhere and indicated that the constructs are independent and cohesive [42, 46]. Both measures showed good internal reliabilities in the present study (Chronbach’s Alphas are reported in Table 1).

**Table 1:** Correlation coefficients between racism experiences and emotional health at Time 1 and 2, and demographic variables measured at Time 1

		1	2	3	4	5	6	7	8	9	10	11	12
1	T1 Direct racism												
2	T1 Vicarious racism	0.304											
3	T1 Loneliness	0.340	−0.029										
4	T1 Depressive symptoms	0.234	−0.119	0.593									
5	T2 Direct racism	0.513	0.290	0.282	0.146								
6	T2 Vicarious racism	0.126	0.419	−0.003	−0.049	0.499							
7	T2 Loneliness	0.396	0.163	0.419	0.335	0.433	0.084						
8	T2 Depressive symptoms	0.391	0.236	0.200	0.214	0.376	0.252	0.695					
9	Female	−0.036	0.131	0.060	0.064	0.099	0.388	−0.092	0.155				
10	Age	0.139	0.117	−0.064	0.186	0.287	0.102	−0.030	−0.042	0.051			
11	Minority English	−0.024	−0.115	0.126	−0.077	0.032	−0.062	−0.025	0.061	0.032	−0.324		
12	Minority non-English	0.232	0.132	0.005	0.125	0.193	−0.052	0.010	0.016	0.175	0.368	−0.456	
Proportion		.32	.73	.25	.39	.35	.75	.30	.32	.47	-	.32	.30
Cronbach’s Alpha		.85	.73	.88	.76	-	-	-	-	-	-	-	-

N = 150; coefficients greater than .16 are significant at *p* < .05

Note: The mean age for the sample was 11.14 (*SD =* 2.20; Range = 8 – 15)

In Australia, self-reported race/ethnicity is not routinely collected at a national or jurisdictional level, neither are race or ethnicity part of the common vernacular. While we recognize that “culture” is not synonymous with race or ethnicity, in Australia, “culture” is commonly used as a proxy for, and conflated with, race and/or ethnicity and/or religion [62]. Thus, in keeping with common vernacular of Australian students, teachers and communities, “culture” was used as a proxy for race/ethnicity within survey items. At the beginning of the survey students were provided the following information “Culture can be about skin colour, language, accent or the country someone is born in. When we say ‘culture’ in these questions we mean any of these things.” In this way students were cued to consider “culture” in relation to what would be considered aspects of race and ethnicity in other country contexts. Similar approaches to measuring racial discrimination have been used in Australian population data in recognition of the unique country context [43]. A specific focus on school as a context for experiences of racism was taken in this study due to school being the most common setting within which children and young people report such experiences occur [34]. Moreover, anti-racism best practice advocates a settings-based approach [39], and this study was ultimately conducted as part of a larger intervention implementation and evaluation as mentioned above.

#### Mental health

Data were collected on two mental health outcomes by asking students to report on single items from the KIDSCREEN-10 scale [49], assessing loneliness (*In the past week I have felt lonely most times, sometimes, not much*) and depressive symptoms (*In the past week I have felt sad most times, sometimes, not much*), each coded as a binary variable for analysis (*most times*/*sometimes* versus *not much*). As previously published [42, 46], despite a high level of association between the two health items (OR 10.19; 95% CI 6.92, 15.02), they demonstrated reasonably low internal consistency as a two-item scale (α = 0.66) and had differential relationships with a number of other variables at a bivariate level. Thus these variables were considered independently in subsequent analyses.

### Data analyses

**Fig. 1:**
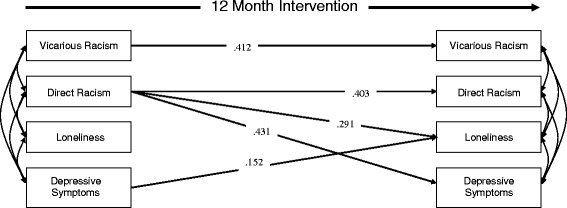
Path analysis testing cross-lagged effects of racism experiences and emotional wellbeing on 1 another over 12 months

To examine relations between different racial discrimination experiences and student mental health, over time we modelled a full cross-lagged panel design using MPLUS v.7 in which all variables were included at both time points. We specified outcome variables as categorical and therefore used a means and variance adjusted weighted least squares estimator. Cross-lagged paths (represented as diagonal lines in Fig. 1) were calculated simultaneously with the direct within-measure longitudinal paths (horizontal lines in Fig. 1). We also included residual correlations between T2 measures (curved pathways between T2 measures on the right hand side of the model in Fig. 1). This provides a more conservative test of cross-lagged effects as it controls for any remaining association between T2 measures. Migrant background/Indigeneity and school were dummy coded and included, along with gender and age, in the model at T1 to account for any variance explained by these demographic factors. Although all demographic variables are included in the regression models they are not presented in the figure for the sake of clarity. This study uses complete case analyses with only participants with data at T1 and T2 included.

## Results

### Relations between different racial discrimination experiences and student mental health, over time

Correlation coefficients between all variables at T1 and T2, as well as demographic differences measured at T1 are presented in Table 1. At the bivariate level, and at both time points, direct racism was positively associated with vicarious racism (*r*_*T1*_ = .304; *r*_*T2*_ = .499), loneliness (*r*_*T1*_ = .340; *r*_*T2*_ = .433) and depressive symptoms (*r*_*T1*_ = .234; *r*_*T2*_ = .376). Vicarious racism was less consistently associated with loneliness (*r*_*T1*_ = −.029; *r*_*T2*_ = .084) and depressive symptoms (*r*_*T1*_ = −.119; *r*_*T2*_ = .252). Finally, loneliness and depressive symptoms were highly correlated at both times (*r*_*T1*_ = .593; *r*_*T2*_ = .695). Standardized and unstandardized probit regression coefficients (along with standard errors and p values for the unstandardized effects) for all longitudinal paths tested in this model are presented in Table 2. Figure 1 presents the significant pathways in this longitudinal model.

**Table 2:** Standardized and unstandardized probit coefficients for the cross-lagged effects of all variables at T1 predicting racism experiences and emotional health at T2. All pathways were estimated simultaneously

	*β*	*b*	*SE*	*p*
Predicting T2 Vicarious racism				
T1 Vicarious racism	0.412	1.139	0.135	0.000
T1 Direct racism	0.085	0.228	0.415	0.582
T1 Loneliness	−0.003	−0.009	0.516	0.986
T1 Depressive symptoms	0.049	0.125	0.396	0.753
Female	0.340	0.841	0.434	0.052
Age	−0.013	−0.007	0.135	0.958
Minority English	−0.221	−0.575	0.241	0.017
Minority non-English	−0.365	−0.969	0.333	0.004
Predicting T2 Direct racism				
T1 Vicarious racism	0.039	0.111	0.306	0.716
T1 Direct racism	0.403	1.123	0.165	0.000
T1 Loneliness	0.122	0.378	0.434	0.385
T1 Depressive symptoms	−0.131	−0.343	0.568	0.546
Female	0.103	0.263	0.402	0.513
Age	0.308	0.178	0.099	0.072
Minority English	0.212	0.571	0.353	0.106
Minority non-English	0.106	0.292	0.345	0.397
Predicting T2 Loneliness				
T1 Vicarious racism	0.074	0.186	0.203	0.359
T1 Direct racism	0.291	0.716	0.221	0.001
T1 Loneliness	0.185	0.507	0.288	0.078
T1 Depressive symptoms	0.152	0.353	0.072	0.000
Female	−0.116	−0.264	0.420	0.531
Age	−0.058	−0.030	0.049	0.541
Minority English	−0.089	−0.212	0.246	0.388
Minority non-English	−0.076	−0.184	0.396	0.642
Predicting T2 Depressive symptoms				
T1 Vicarious racism	0.172	0.451	0.330	0.172
T1 Direct racism	0.431	1.101	0.288	0.000
T1 Loneliness	−0.122	−0.347	0.602	0.565
T1 Depressive symptoms	0.232	0.559	0.685	0.414
Female	0.167	0.391	0.298	0.190
Age	−0.101	−0.053	0.058	0.361
Minority English	0.049	0.121	0.144	0.401
Minority non-English	−0.117	−0.295	0.336	0.380

As shown in Fig. 1, both aspects of racial discrimination experiences exerted a positive auto-regressive effect suggesting a high degree of consistency in direct racism (*β* = .412*, SE* = .135*, p* < .01) and vicarious racism (*β* = .412*, SE* = .135*, p* < .01). In addition, the model included three significant cross-lagged effects in which those reporting direct racism at T1 were more likely to report experiencing loneliness (*β* = .291*, SE* = .221*, p* < .01) and depressive symptoms (*β* = .431*, SE* = .288*, p* < .01) over time. Furthermore, T1 depressive symptoms exerted a modest cross-lagged effect on loneliness (*β* = .152*, SE* = .072*, p* < .01).

As reported in Table 2, direct racism is positively and independently associated with both loneliness and depressive symptoms over time. These associations are unidirectional in that the mental health outcomes do not in turn make students more likely to report experiencing racism. Although T1 mental health was correlated with T2 mental health, these associations appear to be fully explained by the impact of direct racism as there were no autoregressive effects of mental health over time in the full cross-lagged model. Vicarious racism experiences appear to be consistent over time, but were unrelated to emotional health outcomes beyond the impact of direct racism.

We also conducted mediation analyses to formally test whether direct racism experiences explained mental health at T2. A 5,000 boostrap resampling procedure was used to estimate standard errors and 95% confidence intervals (CIs) for the indirect effects. First, T1 depressive symptoms had a significant indirect effect on T2 depressive symptoms via T1 direct racism experiences (*β* = .143, *SE* = .063, 95% CI [.035, .564]), with no remaining direct effect (*β* = ..073, *SE* = .108, 95% CI [−.305, .612]). Likewise, there was a significant indirect effect of T1 loneliness on T2 loneliness via T1 direct racism experiences (*β* = .153, *SE* = .065, 95% CI [.025, .281]). The direct effect of loneliness was also significant (*β* = .269, *SE* = .104, 95% CI [.066, .472]), unlike for depressive symptoms. Thus direct racism fully explained depressive symptoms. Racism experiences also partially explained experiences of loneliness, however loneliness was also partially explained by existing levels of loneliness 12 months prior.

There were few significant cross-sectional associations of demographic variables with either racism experiences or emotional health evident at baseline as reported in Table 1. Minority non-English students were more likely to report direct racism, and older students were more likely to report depressive symptoms. Overall, experiences were relatively stable across demographics. Some differences in vicarious racism experiences by students from minority birth country categories over time were also evident, as reported in Table 2. Specifically, minority non-English students (*β* = −.365*, SE* = .333*, p* < .01) and minority English students (*β* = −.221*, SE* = .241*, p* = .02) were less likely to report vicarious racism at Time 2 compared with majority reference group students.

## Discussion

Perceived experiences of racial discrimination are prevalent and highly salient for children and youth from minority backgrounds [18] with deleterious impacts on numerous concurrent and later outcomes [1, 45]. This study adds to the growing literature investigating the impact of discrimination among children and adolescents outside of the U.S. and from Indigenous and migrant backgrounds. This study extends previous work by showing that perceived direct racial discrimination had significant, negative effects on later depressive symptoms and on later loneliness but that vicarious racial discrimination had no effect on either depressive symptoms or loneliness beyond the effect of direct racial discrimination.

Consistent with existing research citing the direct effects of discrimination on depressive symptoms among adolescents over time [23, 58], the current study found racial discrimination fully explained an increased likelihood of experiencing depressive symptoms over time among Australian students, with no (auto-regressive) effect of T1 depressive symptoms remaining. Also consistent with cross-sectional research [26, 35], racial discrimination exerted a longitudinal effect on loneliness, although the effect size was smaller than for that of racial discrimination on depressive symptoms. Mediation analysis indicated that racism only partially mediated loneliness, which was also directly predicted by existing loneliness 12 months prior. These dimensions of mental health may thus be distinct constructs, at least in terms of how they relate to experiences of racial discrimination [26] in middle childhood and adolescence.

Another distinction between these mental health dimensions in our findings was the cross-lagged (and unidirectional) effect of depressive symptoms on loneliness. Although this effect was not predicted, it may indicate that depressive symptoms are psychologically prior and can lead to loneliness, although other evidence suggests loneliness predicts depression, and that depressive symptoms and loneliness are reciprocally related over time [61]. Our findings are consistent with understandings of depression as an underlying condition, of which loneliness is a more narrow-bandwidth symptom, as operationalized in childhood depression scales that include ‘feeling lonely’ as a symptom [12, 16]. Nevertheless, direct racism was still the most powerful cross-lagged predictor of loneliness in our study, consistent with our expectations.

A recent meta-analysis [50] and empirical evidence [59] indicated reciprocal relationships between peer victimization and psychosocial problems. We found, however, no evidence of reciprocal cross-lagged effects on racial discrimination by either depressive symptoms or loneliness. It is plausible that depressive symptoms and loneliness are differently related to general peer victimization than to racial discrimination as a form of identity-based or bias-based victimization [48], with previous research suggesting general and racial victimization experiences are only moderately correlated among school students [31]. However, the difference in effect sizes for racial discrimination with loneliness and with depressive symptoms in our study are consistent with the findings from the general peer victimization literature that also show victimization is differentially related to specific dimensions of mental health and psychosocial symptoms [59].

Vicarious discrimination was not found to be associated with either depressive symptoms or loneliness over time in this study after accounting for direct racism, suggesting that direct racism is a better predictor of loneliness and depressive symptoms over time than vicarious racism. However, it is also plausible that this finding may be related to methodological issues, and that vicarious experiences of discrimination may be hard to capture or may not have been captured by the measure used in the study. Bivariate correlations between vicarious racism and later loneliness and depressive symptoms are however consistent with studies that show witnessing generalised bullying and harassment impacts negatively on mental health for children and adolescents [36, 51]. More investigation of the experiences and impacts of vicarious discrimination reported by children and adolescents is needed in future studies, both in isolation and in combination with direct experiences of discrimination.

### Limitations

While single-item measures of self-rated health are predictive of objective health outcomes and are widely utilised [13], there is a need to replicate our findings using more robust psychometrically validated instruments of both depressive symptoms and loneliness. Short-form measures were required by the intervention evaluation study from which these data were drawn. However, the current study findings are consistent with other research using multi-item measures of these dimensions of mental health.

Further psychometric validation studies of the racial discrimination measures developed for this study are also required across samples of children from a range of ethnic, migration and language of origin backgrounds. The appropriate conceptualization and measurement of racial discrimination among children, and indeed among adults, and across population groups and contexts, remains under-developed in the field globally, [5, 45].

Other limitations include the need for more detailed measurement of racial/ethnic background, both self-report and socially ascribed, within future research. However, our approach is consistent with the broad analytic categories of ‘immigrant’ or ‘visible minority’ i.e. non-Indigenous non-White/Anglo frequently used in other national settings including Canada [56, 64]. Larger sample size would likely be required for such subgroup analyses, particularly longitudinally. As a community-based study, participants were not randomly selected and it is not a representative population sample. However, the internal validity of the study remains high and many findings are consistent with other research in the field as described earlier. In addition, as noted above, our findings form part of a broader intervention study, elements of which may have influenced variations in prevalence of outcomes over time. Although, separate analyses show a lack of intervention effect over time on student reports of either discrimination or social attitudes [42].

## Conclusions

The current study advances our understanding of how direct and vicarious racial discrimination and loneliness and depressive symptoms are related over time among Australian school students. Future research should continue to examine patterns and impacts of both direct and vicarious experiences of discrimination among children and adolescents in order to help elucidate associations between different experiences of discrimination and child and adolescent health and development outcomes.

## Appendix

**Table 3:** Full list of countries of birth by the analytic categories used

Country of Birth Category	Country	Number of participants reported		
		Students	Fathers	Mothers
Students born in English-speaking countries who also had both parents born in English speaking countries	Australia	51	47	48
	Scotland	2	4	4
	UK		1	
	NZ			1
Students born in English-speaking countries who had one or both parents born in non-English speaking countries	Australia	45	8	11
	NZ	1		
	Argentina		1	
	Cambodia		1	1
	China		1	
	Greece		1	2
	India		1	1
	Iraq		10	10
	Kuwait		2	1
	Lebanon		2	3
	Macedonia		3	
	Malaysia		1	1
	Palestine		1	
	Samoa		3	3
	Serbia		2	1
	Sudan		1	
	Turkey		4	2
	Vietnam		4	4
	Italy			1
	PNG			1
	Philippines			1
	Syria			1
	Taiwan			1
	Thailand			1
Students who were born in non-English speaking countries themselves as well as one or both of their parents	Afghanistan	18	23	23
	Cambodia	1	1	1
	Indonesia	1		
	Iran	1		
	Iraq	3	3	3
	Italy	1	1	1
	Kenya	2		
	Malaysia	1	1	1
	Pakistan	1		
	Philippines	1		1
	RD Congo	1	1	1
	Serbia	2	1	1
	Syria	1	1	
	Sri Lanka	1	1	1
	Sudan	4	3	3
	Thailand	1		1
	Vietnam	1	1	
	Australia		2	2
	Bosnia		1	1
	Ethiopia		1	2
	South Africa		1	1
Students who did not know where one or both of their parents were born or had missing data	Australia	6		
	Iraq	1	1	
	Samoa			1
	China		1	
	Ethiopia		1	
	Don’t know		5	7
	Unreported	1		

## Abbreviations

LEAD: Localities Embracing and Accepting Diversity
LGAs: Local Government Areas
T1: Time 1
T2: Time 2
